# Fabrication of Implantable, Enzyme-Immobilized Glutamate Sensors for the Monitoring of Glutamate Concentration Changes *in Vitro* and *in Vivo*

**DOI:** 10.3390/molecules19067341

**Published:** 2014-06-05

**Authors:** Tina T.-C. Tseng, Cheng-Fu Chang, Wen-Chin Chan

**Affiliations:** 1 Department of Chemical Engineering, National Taiwan University of Science and Technology, Taipei 10607, Taiwan; E-Mail: revive18@gmail.com; 2 Department of Neurosurgery, Taipei Medical University Hospital, Taipei 11031, Taiwan

**Keywords:** glutamate oxidase, chitosan, glutaraldehyde, glutamate, enzyme immobilization, microelectrode, micromachining, rat, hypothalamus

## Abstract

Glutamate sensors based on the immobilization of glutamate oxidase (GlutOx) were prepared by adsorption on electrodeposited chitosan (*Method 1*) and by crosslinking with glutaraldehyde (*Method 2*) on micromachined platinum microelectrodes. It was observed that glutamate sensors prepared by *Method 1* have faster response time (<2 s) and lower detection limit (2.5 ± 1.1 μM) compared to that prepared by *Method 2* (response time: <5 sec and detection limit: 6.5 ± 1.7 μM); glutamate sensors prepared by *Method 2* have a larger linear detection range (20–352 μM) and higher sensitivity (86.8 ± 8.8 nA·μM^−1^·cm^−2^, N = 12) compared to those prepared by *Method 1* (linear detection range: 20–217 μM and sensitivity: 34.9 ± 4.8 nA·μM^−1^·cm^−2^, N = 8). The applicability of the glutamate sensors *in vivo* was also demonstrated. The glutamate sensors were implanted into the rat brain to monitor the stress-induced extracellular glutamate release in the hypothalamus of the awake, freely moving rat.

## 1. Introduction

Glutamate oxidase is one of the key elements when fabricating glutamate sensors. Glutamate oxidase (~140 kDa) has high substrate specificity to glutamate (*k_cat_* = 75 s^−1^ and *K_m_* = 0.23 mM) and high stability (thermal stability ~80 °C) [1,2]. Like other enzyme-based biosensors, glutamate oxidase functions as the biorecognition compound for increasing the sensor selectivity towards glutamate [3]. Glutamate oxidase converts its substrate glutamate in presence of oxygen and water into the products α-ketoglutarate, hydrogen peroxide, and ammonia [4]; the produced hydrogen peroxide can be easily detected amperometrically. However, due to the high cost and the scarcity of commercially available glutamate oxidase, economic and reliable enzyme immobilization methods are demanded for lowering the sensor fabrication cost.

Currently, enzyme immobilization methods for the preparation of electrochemical glutamate sensors including crosslinking [5,6,7,8,9,10], adsorption [11,12], entrapment [13,14], adsorption followed by entrapment [15,16], and coating [17], *etc.* For an ideal immobilization, the immobilized glutamate oxidase needs to have tight binding to the electrode support and also maintain its structure and function after the immobilization. It is worth noting that glutamate oxidase has a unique hexametric α_2_β_2_γ_2_ protein structure and it has two funnel-shaped entrances leading the substrate from the surface to the active site buried deep within the enzyme [1,2]. The most common immobilization method for fabricating glutamate sensors is crosslinking which uses the stabilizing reagent bovine serum albumin (BSA) and the crosslinker glutaraldehyde. Glutaraldehyde is reactive towards the amine groups of lysine residues located mainly on protein surfaces [18], and therefore it can crosslink and immobilize glutamate oxidase and BSA on the electrode surface. Since glutamate oxidase is composed of an oligometric dimer with each subunit containing α-, β-, and γ-fragments [1,2], severe crosslinking may induce conformational changes of the enzyme structure which could possibly lead to a loss of enzyme activity and a decrease in the sensor sensitivity. This crosslinking method is generally simple and straightforward; on the other hand, it requires tedious and skilled labor work. Due to the pH-dependent property of chitosan, the chitosan thin film can be prepared by electrodeposition with good spatial and temporal control [19] and the chitosan thin film has been used to adsorb enzymes and other biomolecules [12,20,21,22,23,24,25,26,27]. The immobilized enzymes may be adsorbed physically on the chitosan surface due to the trapping of enzymes in the chitosan matrix or the electrostatic interaction between the chitosan and enzymes [28]. Since adsorption on chitosan does not involve destructive modification of glutamate oxidase, the loss of enzyme activity may be limited; however, the immobilized enzyme may be desorbed from the electrode support due to the weak binding. Enzyme adsorption using electrodeposited chitosan is a more robotic immobilization method for controlled enzyme immobilization; on the other hand, the amount of surface adsorbed enzyme may be less than that crosslinked in a 3D matrix which can be a cause affects the sensitivity of the biosensor.

Amperometric glutamate sensors can be classified into *first* and *second generation* glutamate sensors based on their electron transfer mechanisms and redox reactions on the electrode [29,30]. For conventional *first generation* glutamate sensors, a one-step redox reaction takes place and only one enzyme (e.g., glutamate oxidase) is involved; the product hydrogen peroxide is oxidized directly at the electrode. However, since the FAD prosthetic group of glutamate oxidase is buried deep inside the protein [1,2], electron transfers could be kinetically slow. For *second generation* glutamate sensors, a two-step reaction is involved and a second redox enzyme (e.g., peroxidase) incorporated with a synthetic redox mediator are used to reduce hydrogen peroxide and replace the direct oxidation of hydrogen peroxide at the electrode [17,31]. In general, *first generation* sensors are simple to fabricate with better reproducibility and usually have faster response time and higher sensitivity; *second generation* sensors could detect analyte at lower potentials and therefore eliminate the interfering current. In this study, two enzyme immobilization methods, physical adsorption with electrodeposited chitosan (*Method 1*) and crosslinking with glutaraldehyde and BSA (*Method 2*), are used to fabricate *first generation* glutamate sensors and analytical figures of merit of these glutamate sensors are compared.

Several studies have demonstrated the utility of implantable glutamate biosensors for the monitoring of behaviorally induced [8,10,32,33], electrically stimulated [8,34], potassium evoked [5,34], and pharmaceutically manipulated [34] glutamate release and locally applied glutamate [5,7,34] in different rat brain areas. The hypothalamus is an important part of the brain that controls endocrine system, body temperature, hunger, sleep, thirst, reproduction, and emotional states. It has been demonstrated that glutamate is the primary endogenous excitatory neurotransmitter released by the hypothalamic neurons [35]. The glutamate sensor has also been used to describe the sleep-awake related glutamate changes in the posterior hypothalamus of freely moving rats [36]. In this study, the stress-induced glutamate release in the rat hypothalamus will be detected using our implantable glutamate biosensors.

## 2. Results and Discussion

### 2.1. Evaluation of Glutamate Sensors Prepared by Method 1 and Method 2

After the sensor fabrication process, glutamate sensors prepared by *Method 1* and *Method 2* were tested *in vitro* to evaluate their analytical figures of merit, including response time, linear range, sensitivity, and limit of detection.

#### 2.1.1. Sensing Current Response

**Figure 1:**
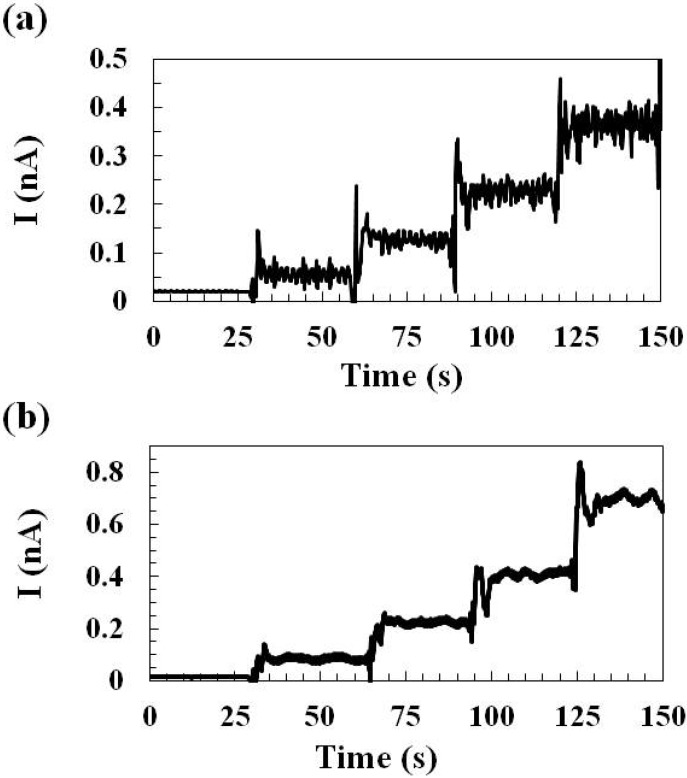
Representative glutamate sensing current responses of sensors prepared by (**a**) *Method 1* and (**b**) *Method 2*. The sensors were tested with different concentrations of glutamate from 0 μM, 20 μM, 60 μM, 119 μM, to 217 μM during which a constant oxidation potential 0.7 V *vs.* Ag/AgCl was applied.

The sensors were tested with different concentrations of glutamate (0 μM, 20 μM, 60 μM, 119 μM, and 217 μM) during which a constant oxidation potential 0.7 V was applied and the glutamate sensing current was recorded (Figure 1). The representative glutamate sensing current responses of sensors prepared by *Method 1* and *Method 2* are shown in Figure 1a,b, respectively. The response time is defined as the sensing current reached 95% of the steady-state current. Based on this definition, it was calculated that the sensor prepared by *Method 1* has the response time <2 s and that prepared by *Method 2* has the response time <5 s. When preparing glutamate sensors by *Method 2*, repeating manual deposition of the crosslinked glutamate oxidase mixture on the electrode surface is required and the resulting enzyme layer is possibly thicker than that prepared by *Method 1* which may consist just a thin adsorbed enzyme monolayer and therefore, glutamate sensors prepared by *Method 2* requires longer time for glutamate to diffuse and reach the electrode surface which could lead to a longer sensor response time.

#### 2.1.2. *In Vitro* Calibration

**Figure 2:**
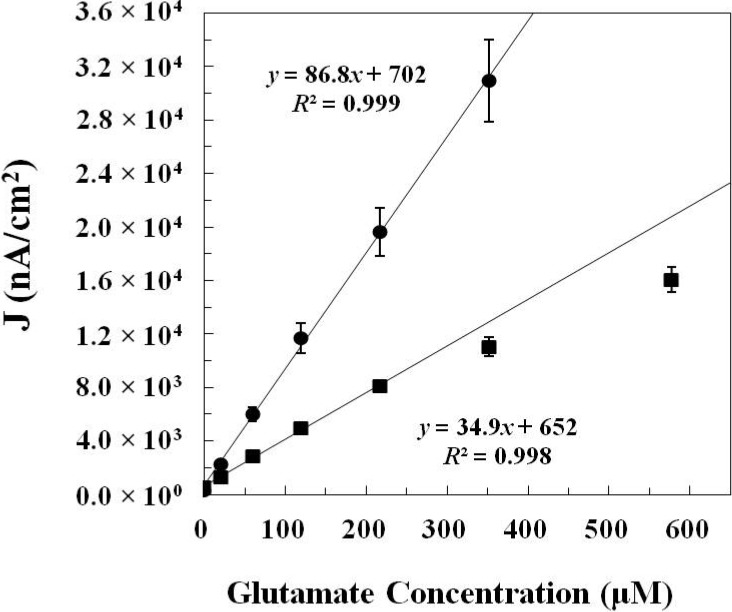
The *in vitro* calibration data of glutamate sensors prepared by *Method 1* (■) (N = 8) and *Method 2* (●) (N = 12). The corresponding glutamate sensing current density (*J* in nA/cm^2^) is plotted *vs.* glutamate concentration (μM).

*In vitro* calibration curves (Figure 2) were obtained by plotting the corresponding glutamate sensing current density (*J* in nA/cm^2^) *vs.* glutamate concentration (μM). For glutamate sensors prepared by *Method 1*, the calibration curve is linear from 20 μM up to 217 μM of glutamate and a trendline (*y* = 34.9*x* + 652, *R*^2^ = 0.998) is obtained by linear regression. Thus, the sensitivity of glutamate sensors prepared by *Method 1* is 34.9 ± 4.8 nA·μM^−1^·cm^−2^ (N = 8). The value of standard deviation is obtained by calculating the standard deviation of sensor sensitivities from eight independent measurements. For glutamate sensors prepared by *Method 2*, the calibration curve is linear from 20 μM up to 352 μM of glutamate and a trendline (*y* = 86.8*x* + 702, *R*^2^ = 0.999) is obtained by linear regression. The sensitivity of glutamate sensors prepared by *Method 2* is 86.8 ± 8.8 nA·μM^−1^·cm^−2^ (N = 12). The limits of detection at two times the level of noise are 2.5 ± 1.1 μM for sensors prepared by *Method 1* and 6.5 ± 1.7 μM for sensors prepared by *Method 2*. The summary of figures of merit of glutamate sensors prepared by *Method 1* and *Method 2* is shown in Table 1. Although fabricating sensors by *Method 2* (crosslinking) may change the conformation of the enzyme structure and decrease the activity of the immobilized enzyme which causes a decrease in the sensor sensitivity, the repetitive manual deposition of high units of glutamate oxidase could possibly increase the enzyme loading on the electrode and therefore, glutamate sensors prepared by *Method 2* still have higher sensor sensitivity than that prepared by *Method 1*.

**Table 1:** Figures of merit of glutamate sensors prepared by *Method 1* and *Method 2*.

GlutOx Immobilization Method	Linear Range (μM)	Sensitivity (nA·μM^−1^·cm^−2^)	Limit of Detection (μM)	Response Time (s)
Adsorption with electrodeposited chitosan (*Method 1*)	20–217	34.9 ± 4.8 (N = 8)	2.5 ± 1.1	<2
Crosslinking with BSA and glutaraldehyde (*Method 2*)	20–352	86.8 ± 8.8 (N = 12)	6.5 ± 1.7	<5

#### 2.1.3. Effect of Interference and Sensor Stability

**Figure 3:**
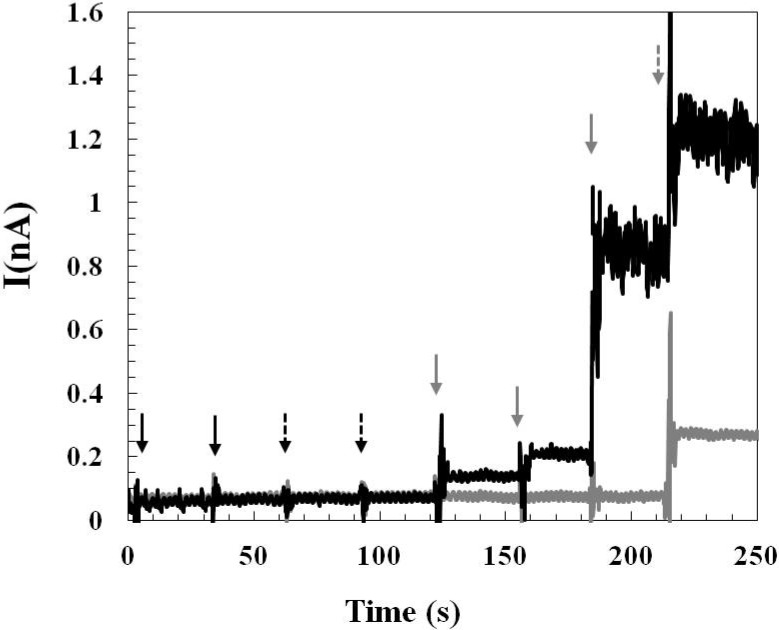
A representative result of sensing current responses by testing the glutamate sensor prepared by *Method 2* (▬ *black line*) and the control sensor (▬ *gray line*) with dopamine (5 μM and 10 μM), ascorbic acid (250 μM and 500 μM), glutamate (20 μM, 40 μM, 240 μM), and hydrogen peroxide (20 μM), sequentially. The injection of dopamine is indicated by the *solid black arrow*, the injection of ascorbic acid is indicated by the *dashed black arrow*, the injection of glutamate is indicated by the *solid gray arrow*, and the injection of hydrogen peroxide is indicated by the *dashed gray arrow*. The control sensor was prepared by the same sensor fabrication process as that of the glutamate sensor except that the control sensor does not have the enzyme layer.

The examination of the effect of interference from common electroactive interferents presenting in the central nervous system is an important checkpoint before conducting *in vivo* experiments. Glutamate sensors prepared by both methods showed good selectivity towards glutamate and against negatively charged ascorbic acid and positively charged dopamine. A representative result of sensing current response by testing the glutamate sensor (prepared by *Method 2*) with dopamine, ascorbic acid, glutamate, and hydrogen peroxide is shown in Figure 3. No increase in sensing current steps was observed when testing the sensor with dopamine (5 μM and 10 μM) and ascorbic acid (250 μM and 500 μM). The glutamate sensing current increased as expected when the glutamate concentration increased from 20 μM, 40 μM, to 240 μM. Both the glutamate sensor and the control sensor responded to hydrogen peroxide (20 μM) indicating that the platinum microelectrodes functioned normally. Although glutamate sensors prepared by *Method 1* and *Method 2* are both *first generation* sensors which oxidize hydrogen peroxide at high oxidation potential (+0.7 V), the results of the interference test suggest that the problem of oxidizing/reducing electroactive interfering species could be overcome by modifying the electrode with pretreatment layers (polypyrrole and Nafion^®^) and good sensor selectivity was achieved. 

The storage stability of the glutamate sensor has been investigated by testing sensor sensitivities everyday for a 9-day period (sensors were stored in a desiccated box at 4 °C). A better sensor storage stability was observed from the sensor prepared by *Method 1* which retains 70% of its original activity after 9 days and its loss in activity may due to the desorption of the enzyme from the chitosan deposited electrode surface. The sensor prepared by *Method 2* retains 50% of its original activity after 9 days and its severe loss in activity over time may due to the thick enzyme layer peeling off the electrode surface because of the thermal contraction/expansion effect.

### 2.2. The Applicability of the Glutamate Sensors for in Vivo Study

In order to demonstrate the applicability of the glutamate sensors for monitoring glutamate release *in vivo*, sensors were implanted in the rat brain and tail-pinch stressors were administrated to induce glutamate concentration changes in the hypothalamus of the awake, freely moving rat.

#### 2.2.1. Pre-Calibration and Post-Calibration

**Figure 4:**
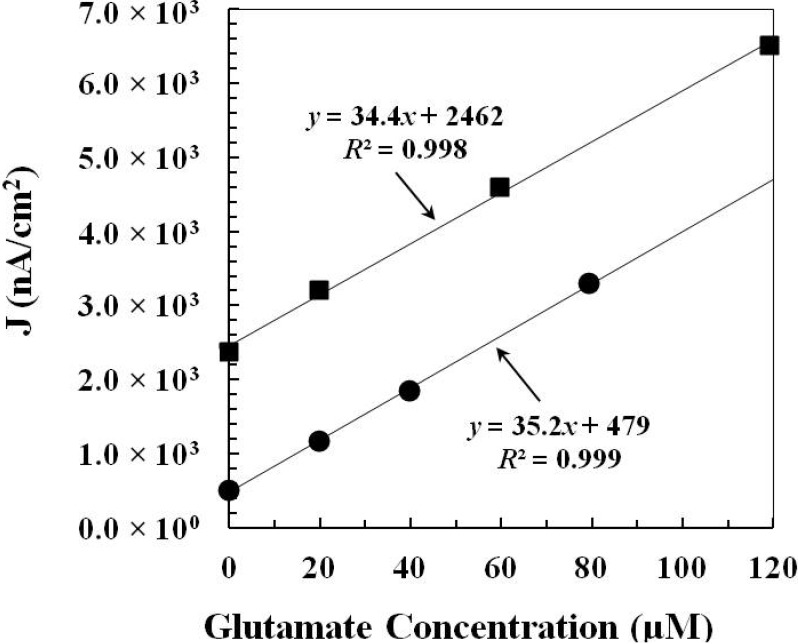
The pre-calibration data (■) and post-calibration data (●) of the glutamate sensor (prepared by *Method 1*) before and after the *in vivo* study. The corresponding glutamate sensing current density (*J* in nA/cm^2^) is plotted *vs.* glutamate concentration (μM).

When applying biosensors for *in vivo* experiment, biofouling of sensor surfaces with endogenous biomolecules is a common problem resulting in the decrease of sensor sensitivity. The sensitivity of the glutamate sensor was examined fifteen minutes before and fifteen minutes after the *in vivo* experiment. The pre-calibration and post-calibration curves of the glutamate sensor (prepared by *Method 1*) before and after the *in vivo* study were shown in Figure 4. 

Calculated from the calibration data, the sensitivities of the glutamate sensor before and after the *in vivo* experiment are 34.4 nA·μM^−1^·cm^−2^and 35.2 nA·μM^−1^·cm^−2^, respectively. The calibration result demonstrates that the glutamate sensor can retain its good sensitivity before and after the *in vivo* experiment and thus, the biofouling on the glutamate sensor surface during the *in vivo* experiment may not be a problem. It is possible that the protection layer coated outside the enzyme layer secures the immobilized enzyme and prevents the enzyme loss during the sensor implantation process. Besides, this result also confirms that the enzyme modified layer of the glutamate sensor still retains its original function after the implantation process.

#### 2.2.2. Monitoring Glutamate Release in the Rat Hypothalamus

**Figure 5:**
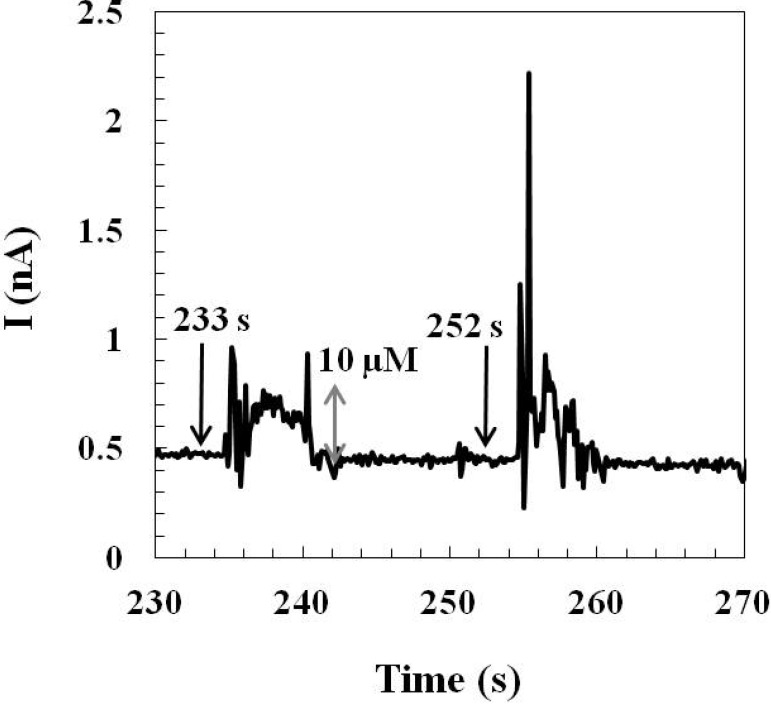
A representative result of the stress-induced glutamate sensing current corresponding to the glutamate release in the hypothalamus of an awake, freely moving rat. Arrows indicate the timing of each tail pinch. A calibration bar (*gray double-sided arrow*) is shown on the plot. The calibration bar was determined by the slope of the post-calibration curve of each implanted glutamate sensor.

The pre-calibrated glutamate sensor (prepared by *Method 2*) was implanted in the rat hypothalamus for monitoring the stress-induced glutamate concentration changes in the awake, freely moving rat. Instant tail pinches (0.5~1 s per pinch) were used as stressors to induce glutamate releases in the hypothalamus. The glutamate sensing current was recorded over time during the tail-pinch course (Figure 5). 

An immediate glutamate current response was observed right after each tail pinch. Each evoked sensing current reached a maximum sensing current in 2–3 s and each current response lasted for 5–6 s. The 2–3 s duration before the sensing current approaching the maximum may count the response time of the glutamate sensor as well as the time required for the input of afferent signals to the central nervous system and the neuronal glutamate release in the hypothalamus. Other previously published studies also reported the application of implantable, enzyme-based *first generation* glutamate sensors for recording behaviorally induced glutamate concentration changes by tail-pinch stress in different brain areas of freely moving rats [8,32,33]. Lowry *et al.* used a Pt wire-based glutamate sensor to detect extracellular glutamate changes in the striatum by applying a 10 s tail-pinch stressor and they observed a sensing current plateau in 3 s which corresponded to a change of 0.5 μM glutamate [32]. Rutherford *et al.* performed experiments of 5 min tail-pinch induced stress to measure changes in glutamate level in the right striatum using ceramic-based microelectrode arrays glutamate sensors [33] and a bimodal glutamate response, a rapid large spike followed by a prolonged glutamate changes, was observed. In our studies of tail-pinch induced stress, we also observed similar biphasic glutamate responses when a much shorter tail pinch (0.5~1 s) was applied: the initial phase was a ~1 s rapid spike and the second phase of the response lasted 5–6 s with a gradual return to the baseline level. Similar studies of monitoring tail-pinch induced glutamate changes in the dorsal striatum using silicon-based microelectrode array biosensors had been reported by Wassum *et al.* and they observed 2.9 μM and 4.9 μM glutamate concentration changes evoked by 1 s tail pinches [8]. This work demonstrates the applicability of our micromachined glutamate sensors for the detection of behaviorally induced transient glutamate concentration changes in a specific brain area, especially in the deep region of the rat brain, hypothalamus.

## 3. Experimental Section

### 3.1. Materials

Bovine serum albumin lyophilized powder (BSA, purity: 96%), chitosan (from shrimp shells, ≥75% deacetylated), l-glutamic acid (Glut, purity: 99%), hydrogen peroxide solution (30%), and glutaraldehyde solution (25 wt % in H_2_O) were purchased from Sigma-Aldrich (St. Louis, MO, USA). Pyrrole (purity: 99%), Nafion^®^ (5%), Drierite^®^, and dopamine hydrochloride (DA, purity: 99%) were purchased from Acros (Geel, Belgium, EU). l-Ascorbic acid (AA, purity: 97%) was purchased from Enzo Life Sciences (Farmingdale, NY, USA). 2-Propanol (IPA, purity: 99.8%) was purchased from Panreac (Montcada i Reixac, Spain) and sulfuric acid 2 N solution was purchased from Kanto Chemical Co., Inc. (Chuo-ku, Tokyo, Japan). Glutamate oxidase (EC 1.4.3.11, oxygen oxidoreductase deamiating; form: α_2_β_2_γ_2_ with α-fragment 43 kD; β-fragment 17 kD; γ-fragment: 10 kD) from *E. coli* recombinant was purchased from US Biological (Marblehead, MA, USA). Ag/AgCl glass-bodied reference electrodes with 3 M NaCl electrolyte and a 0.5 mm diameter platinum (Pt) wire auxiliary electrode were purchased from ALS Co., Ltd. (Tokyo, Japan). Sodium phosphate buffer (PBS) was composed of 50 mM sodium phosphate (dibasic) and 100 mM sodium chloride (pH 7.4).

### 3.2. Instrumentation

A versatile multichannel potentiostat (model VSP300) equipped with the “p” low current option driven by EC-LAB software (Bio-Logic USA, LLC, Knoxville, TN, USA) was used for both *in vitro* electrochemical experiments including sensor preparation, evaluation, and calibration which were performed in a three-electrode configuration consisting of a working electrode, a Pt wire auxiliary electrode, and a Ag/AgCl glass-bodied reference electrode and *in vivo* electrochemical measurements in a two-electrode configuration consisting of a working electrode and a Ag/AgCl wire (200 μm) reference electrode.

### 3.3. Preparation of Micromachined Microelectrodes

The micromachined microelectrodes were fabricated at the Nano-Electro-Mechanical-Systems (NEMS) Research Center, National Taiwan University (Taipei, Taiwan), the National Nano Device Laboratories (NDL, Hsinchu and Tainan, Taiwan), the Nano Facility Center (NFC), National Chiao Tung University (Hsinchu, Taiwan). The fabrication process included general micromachining processes, such as thermal oxidation of a four-inch silicon wafer (200 ± 20 μm in thickness) for the formation of field oxide insulation layer, photolithography for defining the pattern of microelectrodes, for defining the etching area, and for defining the outline of the probe device, metal deposition of a thin layer of platinum film (~100 nm) for the formation of microelectrodes, plasma-enhanced chemical vapor deposition for the formation of insulation layers, and etching process for exposing the electrode sites and the bonding pads from the insulation layer and for etching the outline of the probe device. Each probe device consists of four platinum microelectrodes (each microelectrode has area ~4.490 × 10^−5^ cm^2^) located at the end of the probe shaft (the length and the thickness of the shaft are 9 mm and ~200 μm, respectively) and four connections on the shaft which connect the microelectrodes and bonding pads. Resulting probe devices were then packaged by soldering copper wires to the bonding pads and then protecting the soldering connections with epoxy.

### 3.4. Fabrication of Glutamate Sensors

The microelectrodes are cleaned with IPA under sonication for at least 3 min and then rinsed with deionized water and blow-dried with argon before use. The microelectrodes are first pretreated with electrodeposited polypyrrole for blocking electroactive interferents, such as dopamine and ascorbic acid [37]. Pyrrole monomer (200 mM) is dissolved in SPB and electrodeposited on the microelectrodes by applying 0.85V oxidation potential for 5 to 6 min under stirring condition. Then, the microelectrodes are dip-coated with 5% Nafion^®^ and dried in the convection oven (180 °C) for 3 min (this process is repeated for one more time). The Nafion^®^ layer is used to block negatively charged interferents, such as ascorbic acid and uric acid [38]. After the pretreatment process, a pretreatment layer was formed on the platinum electrode and the microelectrodes are ready for GlutOx immobilization.

**Figure 6:**
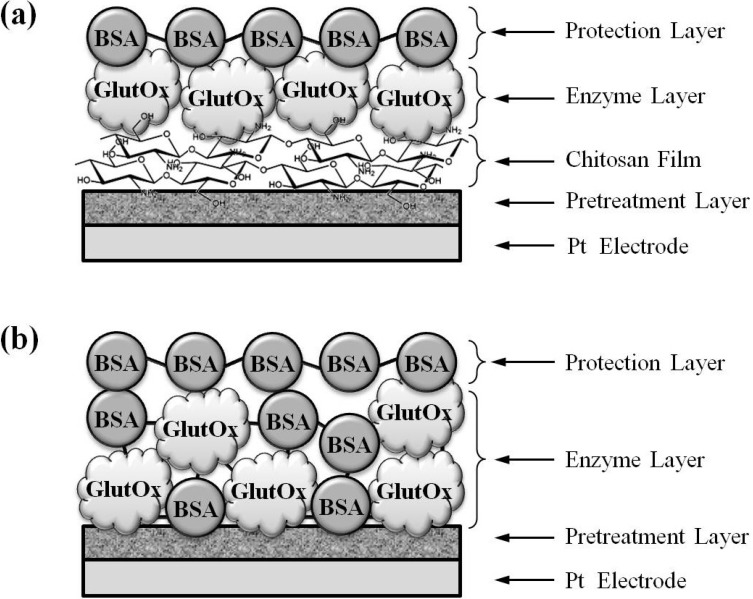
The schematic configuration of the glutamate sensor prepared by (**a**) *Method 1* (immobilization of glutamate oxidase by adsorption on the electrodeposited chitosan) and (**b**) *Method 2* (immobilization of glutamate oxidase by crosslinking with the stabilizing reagent BSA and the crosslinker glutaraldehyde).

Immobilization of GlutOx by *Method 1* (adsorption on the electrodeposited chitosan). Due to the pH-dependent solubility of chitosan (the pKa of the primary amine groups of chitosan is ~6.5), a reduction potential is applied to create a locally high pH near the electrode surface and chitosan becomes insoluble under this condition and therefore, the chitosan film can be electrodeposited on the electrode surface [28]. Electrodeposited chitosan was prepared by applying a constant reduction potential at −1.0 V on the microelectrodes in 0.04% chitosan solution (pH = 5) under stirring condition for 120 s for 3 times [12]. After rinsing the microelectrodes with deionized water and blowing dry the surface with argon, the microelectrodes deposited with chitosan were soaked in 250 unit/mL glutamate oxidase solution for 8 h at 4 °C. Finally, a protection layer was coated atop the enzyme layer to secure the immobilized enzyme and to prevent the enzyme loss during the implantation process. The protection layer was deposited by dip-coating 3 layers of crosslinked BSA on the electrode surface with a glutaraldehyde mixed BSA solution (the solution is a mixture of 10 mg BSA and 5 μL glutaraldehyde (25%) in 1 mL deionized water). The resulting glutamate sensors were stored in an airtight and desiccant container and stored at 4 °C before use. The schematic configuration of the glutamate sensor prepared by *Method 1* is shown in Figure 6a.

Immobilization of GlutOx by *Method 2* (crosslinking with glutaraldehyde). A glutaraldehyde mixed BSA solution (same composition as described previously) was first prepared. GluOX solution (250 unit/mL) and the mixed BSA solution were blended in 1:1 volumetric ratio. The blended enzyme solution was used for coating the enzyme on the microelectrodes by repeating manual deposition for 40 times with a 5 μL syringe under the microscope. Waiting until the enzyme coating was dried (~10 min), the microelectrodes were dip-coated with 3 layers of crosslinked BSA with a glutaraldehyde mixed BSA solution (same composition as described previously). The resulting glutamate sensors were stored in an airtight and desiccant container and stored at 4 °C before use. The schematic configuration of the glutamate sensor prepared by *Method 2* is shown in Figure 6b.

### 3.5. Preparation of Ag/AgCl Wire Reference Electrodes for in Vivo Measurements

A piece of Teflon^®^ coated silver wire (2 cm long and inner diameter ~200 μm) was soldered to the copper wire and the joint was covered with epoxy for insulation. The Teflon^®^ coating on the other end of the silver wire was removed for 1 mm and the exposed part of the silver wire was soaked in 0.1 M hydrochloride solution. A triple-A battery was used for the electrodeposition of AgCl during which the silver wire was the anode and a platinum wire was used as the cathode. The resulting Ag/AgCl wire reference electrode was stored in 1 M potassium chloride solution before use.

### 3.6. Implantation of Glutamate Sensors into the Rat Brain

Adult male Sprague-Dawley rats (~400 to 500 g) were used as the subjects. Animals were anesthetized using 4% chloral hydrate (~1 to 1.5 mL per 100 g rat) by intraperitoneal injection before the surgery. Standard stereotaxic surgical techniques were used to unilateral implant a pre-calibrated glutamate biosensor into the hypothalamus using the following coordinates relative to bregma (AP: −1.8 mm, ML: −0.4 mm, DV: −9.0 mm) according to the atlas of Paxinos and Watson (6th edition) [39]. A previously prepared Ag/AgCl wire reference electrode (200 μm in diameter) was implanted contralaterally. Three small screws were inserted into the skull to float the dental cement for securing the sensor and the reference electrode. Once the dental cement was dried, the skin was sutured and only the electric connection wires of the sensor and the reference electrode were left outside. After the surgery, the rats were allowed to recover from anesthesia on the heating pad for 30 min and then transferred to individual cages with food and water available *ad libitum*. The *in vivo* experiments were carried out one day after the sensor implantation.

### 3.7. In Vivo Experiments

For *in vivo* measurements, electrochemical experiments were performed in a two-electrode configuration consisting of a glutamate sensor working electrode and an Ag/AgCl wire reference electrode. The rat was able to move freely in a box within a Faraday cage. At test, the electric wires of the implanted glutamate sensor and reference electrode were connected to the potentiostat and a constant potential +0.7 V was applied to the working electrode for monitoring the corresponding glutamate sensing current. Once the background current of the glutamate sensor became stable (~15 min equilibrium time), the experimentation is allowed to start. Instant tail pinches (0.5~1 s per pinch) were administrated using stainless forceps. The monitoring current signal was recorded *versus* time and the current changes can be converted to the corresponding glutamate concentration changes according to the post-calibration data.

## 4. Conclusions

Figures of merit of glutamate sensors prepared by two different enzyme immobilization methods, adsorption on the electrodeposited chitosan (*Method 1*) and crosslinking with glutaraldehyde (*Method 2*), were compared. Glutamate sensors prepared by *Method 1* have faster response time and lower detection limit compared to that prepared by *Method 2*. Glutamate sensors prepared by *Method 2* have larger linear detection range and higher sensitivity compared to that prepared by *Method 1*. The *in vivo* study demonstrates the applicability of the implantable glutamate sensors for the detection of the transient stress-induced glutamate release in the rat hypothalamus of the awake, freely moving rat.
